# Frailty and Cardiometabolic Outcomes: A Narrative Review

**DOI:** 10.3390/jcm15041348

**Published:** 2026-02-09

**Authors:** Saam Foroshani, Kevin S. Tang, Nathan D. Wong

**Affiliations:** 1 Department of Medicine, University of California Irvine Medical Center, Orange, CA 92868, USA; foroshas@hs.uci.edu; 2 Heart Disease Prevention Program, Mary & Steve Wen Cardiovascular Division, University of California Irvine, Irvine, CA 92697, USA; ndwong@hs.uci.edu

**Keywords:** frailty, cardiovascular disease outcomes, cardiovascular risk assessment

## Abstract

Frailty is a multidimensional state of reduced physiological reserve that is increasingly recognized as a major determinant of outcomes in cardiovascular diseases (CVDs). As populations age and cardiometabolic multimorbidity becomes more prevalent, understanding how frailty interacts with CVD pathology has important implications for risk stratification, clinical decision-making, and patient-centered care. Across diverse cardiovascular conditions and interventions, frailty independently predicts higher risks of mortality, major adverse cardiovascular events (MACE), rehospitalization, procedural complications, functional decline, and reduced quality of life. Shared biological mechanisms—including chronic inflammation, sarcopenia, endothelial dysfunction, and the effects of multimorbidity and polypharmacy—help explain the strong and often bidirectional relationship between frailty and CVD, one that is reported by recent data to be multiplicative as well as additive. Importantly, frailty demonstrates prognostic value beyond traditional risk factors and varies in predictive performance depending on the assessment tool used. Finally, frailty should not be viewed as immutable; evidence shows that appropriate conditioning may slow the decline or even reverse frail or prefrail states. This narrative review aims to synthesize contemporary evidence on frailty definitions and assessment, epidemiology, mechanistic pathways linking frailty with CVD, associated outcomes, prognostic value, and emerging interventions relevant to CVD prevention and management.

## 1. Introduction

With progressive population aging, the global burden of cardiovascular diseases (CVD) and CVD multimorbidity has increased significantly in the preceding decades, a trend that is expected to continue well into the coming years [1,2]. The interaction between CVD and frailty, a geriatric syndrome defined as the systemic age-related decline in physiologic reserve, is increasingly recognized as an important factor in risk prognostication [3,4]. In high-income countries, the pooled prevalence of frailty in older adults is estimated at around 10% and has been linked with higher rates of hospitalization, falls, and increased healthcare costs [5,6,7].

Frailty is commonly understood through two main frameworks: as a clinical syndrome, or as a state of accumulated health deficits [8,9]. The syndrome-based model, best represented by the Fried frailty phenotype, links frailty to impaired stress tolerance and changes in metabolism [10,11]. It is defined by a cluster of features—exhaustion, weakness, reduced walking speed, low physical activity, and weight loss [12]. An individual with none of these features is considered not frail, those with one or two are prefrail, and those with three or more are considered frail. Meeting all five features significantly increases risk for morbidity and signifies a limited potential for recovery [13].

The deficit-accumulation model defines frailty as the progressive build-up of age-related health problems across multiple domains [9,14]. These deficits may include chronic diseases, functional or cognitive decline, disability, malnutrition, and even abnormal laboratory findings, depending on the data sources used—such as clinical records, geriatric assessments, or surveys. This framework uses the “frailty index,” which represents the ratio of observed deficits to the total number assessed, typically requiring at least 30 items [9]. Higher scores represent greater vulnerability, and values above 0.70 are rare but signal a deficit load that is nearly incompatible with long-term survival. Although no single definition has achieved universal agreement, both this index-based approach and the Fried phenotype model remain widely applied in research and clinical practice [8]. Both conceptual frameworks of frailty are compared in Table 1.

Frailty has a notable relevance in CVD, where the physiologic vulnerability it reflects can compound the burden of aging and chronic illness. Both models of frailty highlight diminished reserve and impaired adaptability, factors that parallel the progression of CVD and CVD multimorbidity. Several studies have demonstrated that frail individuals with CVD often experience worse outcomes, including higher rates of hospitalization, procedural complications, and mortality, underscoring the importance of incorporating frailty into routine cardiac care [15]. This review aims to provide a comprehensive overview of frailty assessment, proposed biologic mechanisms of frailty and its relation to CVD outcomes, and emerging evidence on frailty reversal and its role in clinical practice.

## 2. Epidemiology of Frailty and Cardiovascular Disease

Frailty and prefrailty are highly prevalent among older adults, with pooled estimates from population-based studies indicating a prevalence of frailty around 12% (physical phenotype) to 24% (deficit accumulation model), and pre-frailty affecting around 39% of older individuals [16]. The higher prevalence of frail adults using the frailty index has been reported in other population-level studies and is postulated to be due to the index’s granularity and improved sensitivity for detecting prefrail and mildly frail states [8,17]. Frailty prevalence is even higher in institutionalized and hospitalized patients, with some estimates reaching above 40% [18]. Prevalence increases with age and is consistently higher in women, although one meta-analysis showed that older frail women generally had better survival rates when compared to older frail men [19,20]. Multidimensional frailty is more common in institutionalized settings (such as nursing homes or long-term acute care facilities), but even in community-dwelling populations, it remains a major public health concern [16].

Frailty frequently co-occurs with CVD. Cross-sectional and longitudinal studies show that the presence and number of cardiometabolic conditions—including ischemic heart disease, stroke, diabetes, and hypertension—are strongly associated with increased frailty severity [21]. For example, the frailty index rises incrementally with each additional CVD diagnosis, with stroke exerting the greatest impact on frailty burden [22]. Metabolic syndrome is independently associated with both prevalent and incident frailty, as measured by both the Fried phenotype and frailty index [23]. Furthermore, both baseline frailty and frailty progression have been demonstrated to be independently associated with increased incidence and progression to advanced cardiovascular-kidney-metabolic (CKM) syndrome [24].

**Figure 1:**
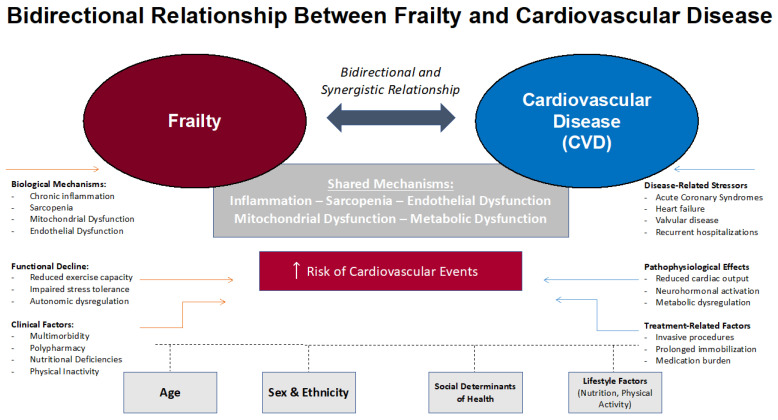
The Bidirectional Relationship Between Cardiovascular Disease and Frailty.

There is robust evidence for a bidirectional association between frailty and CVD, portrayed in Figure 1. Mendelian randomization studies confirm that genetic liability to frailty increases the risk of coronary artery disease, stroke, and type 2 diabetes, while genetic predisposition to these conditions also increases frailty index scores [25]. Prospective cohort and meta-analytic data further demonstrate that frailty and pre-frailty independently predict incident cardiovascular disease, major adverse cardiovascular events, and cardiovascular mortality, even after adjustment for traditional risk factors [26,27,28]. Conversely, the presence of CVD accelerates the progression and severity of frailty, with improvement in frailty status being associated with reduced risk of incident cardiovascular disease and mortality [27].

Shared pathophysiological mechanisms—including chronic inflammation, metabolic dysregulation, and vascular dysfunction—underlie the interplay between frailty and CVD, amplifying vulnerability and adverse outcomes in older adults [29,30]. These findings underscore the importance of routine frailty screening and targeted interventions in populations at risk for or living with CVD.

## 3. Mechanistic Pathways Linking Frailty and Cardiovascular Outcomes

Frailty and adverse cardiovascular outcomes are linked by shared biological mechanisms that accelerate functional decline and vulnerability. Chronic low-grade inflammation (“inflammaging”) is a central driver, promoting catabolism, sarcopenia, and endothelial dysfunction, and is associated with elevated cytokines (e.g., IL-6, TNF-alpha) and immune dysregulation in both frailty and CVD. This inflammatory state impairs muscle protein synthesis, increases oxidative stress, and contributes to atherosclerosis and insulin resistance [31,32]. Vascular endothelial dysfunction shares a similar pathophysiology rooted in excess inflammation, and several recent studies have noted a concurrent association between vascular endothelial dysfunction and frailty, although the exact mechanism underlying this association remains unclear and is a focus for future research [32,33].

Ultimately, impaired muscle protein synthesis secondary to the above mechanisms leads to sarcopenia, which is defined as loss of skeletal muscle mass and function. This is a characteristic feature of physical frailty and is highly prevalent in heart failure and other cardiac conditions, amplifying risk for disability and mortality. Increased sarcopenia and decreased functional capability consequently lower aerobic exercise capacity and contribute to the progression of CVD in frail individuals [8]. Mechanistically, muscle breakdown in sarcopenic patients occurs as a result of declining anabolic hormones and an increase in catabolism [8].

Polypharmacy and multimorbidity are common in frail older adults and exacerbate cardiovascular risk by increasing the likelihood of adverse drug reactions, drug-disease interactions, and cumulative physiological stress. Indeed, the polypharmacy problem is pervasive across older adults, with some estimates placing the average number of daily pills in octogenarians at greater than nine [34]. One study of 250 predominantly older adults hospitalized for CVD found that over 98% of patients experienced at least one drug–drug interaction, with 35% of patients exhibiting 3 or more interactions [35]. The American Heart Association further highlights that frailty amplifies hospital length of stay and adverse outcomes in patients with CVD, especially in the context of multimorbidity and complex medication regimens [6,36]. Furthermore, certain nutritional deficiencies (e.g., vitamin D, calcium) and protein-energy malnutrition are modifiable risk factors for both frailty and CVD, contributing to sarcopenia, bone fragility, and impaired immune function [3]. Conversely, non-modifiable risk factors, such as sex and racial/ethnic differences influence vulnerability to frailty and cardiovascular outcomes. Sex-specific differences in frailty prevalence and mortality risk have even been colloquially referred to as the “sex-frailty paradox.” [37]. The exact mechanisms behind women’s propensity towards increased frailty prevalence while maintaining a lower mortality rate than their male counterparts remain uncertain, although molecular profiling has identified sex-specific mitochondrial signatures in frailty, and variations in types of chronic diseases constituting morbidity burden across sexes may modulate risk [33,37,38].

**Figure 2:**
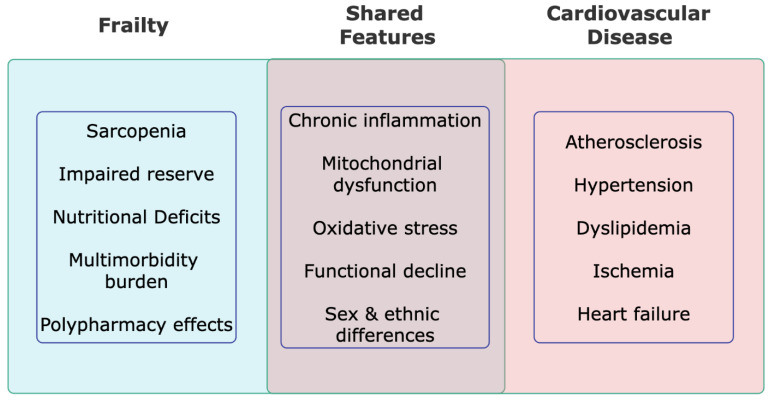
Individual and Shared Features of Frailty and Cardiovascular Disease.

Overall, frailty and cardiovascular disease share similar biological pathways (e.g., chronic inflammation, sarcopenia, mitochondrial and endothelial dysfunction) exacerbated by polypharmacy, nutritional deficits, and multimorbidity with important sex and ethnic differences in susceptibility and outcomes, as portrayed in Figure 2.

## 4. Clinical Outcomes Associated with Frailty in Cardiovascular Disease

Frailty is a well-established independent predictor of both all-cause and CVD mortality [39]. Large-scale cohort studies, including analyses of over 3 million United States Veterans, have demonstrated a strong, graded association between frailty severity and CVD mortality, myocardial infarction, and stroke, independent of underlying CVD and traditional risk factors [40]. One prospective cohort study of over 4000 Medicare patients found frailty to be associated with increased risk of all CVD, reporting hazard ratios (HRs) of 1.77, 1.95, 1.71, 1.80, and 1.35 for MACE, acute myocardial infarction, stroke, peripheral vascular disease, and coronary artery disease, respectively [15]. Another recent analysis of nearly half a million adults from the UK Biobank found the presence of frailty to produce a multiplicative effect with co-occurring CVD on the rates of all-cause and CVD mortality [41]. Furthermore, those with concomitant frailty and cardiometabolic multimorbidity (CMM) exhibited significantly elevated risk of both all-cause (HR 4.91) and CVD mortality (HR 8.33), with substantial portions of this excess risk (23% and 36%, respectively) attributable to additive interaction [41,42]. It should be noted that subjects from this database were generally younger and healthier and may not be generalizable to older or sicker populations [42], however the consistent and significant associations between frailty and increased CVD morbidity and mortality across various geographies and patient demographics offer strong evidence that such additive effects are ubiquitous.

In individuals indicated for cardiovascular intervention, frailty consistently predicts worse outcomes with the magnitude of risk varying by procedural category. Meta-analyses of percutaneous coronary intervention (PCI) and cardiac surgery show that frail patients have significantly higher short-, mid-, and long-term mortality (HRs ~2.2–3.9) and greater risk of myocardial infarction (MI), stroke, heart failure hospitalization, and major bleeding [26,39,43,44]. In acute coronary syndromes, higher frailty scores also correlate with increased in-hospital complications, including stroke, bleeding, vascular injury, and death, while observational data show that frailty prevalence among older adults with acute MI undergoing PCI may exceed 40% [45,46,47]. Although invasive therapy can still offer benefit even in frail subgroups, as illustrated by a 41% reduction in mortality with PCI in frail patients with non-ST elevation MI (NSTEMI), frailty uniformly predicts higher procedural complications including acute kidney injury, perioperative stroke, and prolonged hospitalization after PCI, coronary artery bypass graft (CABG), and vascular interventions [15,39,43,45,48].

Across structural heart interventions, the relative effect of frailty is particularly pronounced. While frailty confers modest increases in mortality after CABG or surgical valve procedures (ORs ~1.1–2.6), its impact is substantially greater in transcatheter aortic valve replacement, where it is associated with a three- to fivefold increase in mortality, major adverse cardiovascular events, and functional decline [49]. Frailty also predicts greater healthcare utilization, including approximately double the risk of rehospitalization, longer hospital stays, and increased post-acute care needs, even after accounting for comorbidities and procedural risk [43,47,49]. These findings underscore the importance of integrating frailty assessment into cardiovascular risk stratification and procedural decision-making.

Finally, frailty is a major driver of functional decline and reduced quality of life in cardiovascular disease. Frail patients experience greater impairments in physical functioning, cardiac symptoms, and health-related quality of life, with standardized mean differences indicating substantial decrements compared to non-frail peers [43,50]. In heart failure and valvular disease, frailty is associated with worse baseline health status and increased risk of subsequent deterioration, although interventions such as cardiac rehabilitation may yield meaningful improvements even in frail individuals [50]. The overlap between frailty and CVD highlights the need for integrated management strategies that address both physiological reserve and disease-specific health status.

## 5. Prognostic Value of Frailty

Frailty has emerged as a powerful independent predictor of adverse outcomes in patients with cardiovascular disease, extending beyond what can be explained by traditional risk factors such as age, hypertension, diabetes, and smoking. Unlike isolated comorbidities, frailty captures the cumulative vulnerability of multiple organ systems and the diminished physiologic reserve that characterizes many older adults. As Kim and Rockwood emphasize, frailty reflects the biological rather than chronological dimension of aging, serving as a translational measure of health that can meaningfully stratify risk where conventional markers fall short [8]. Several large cohort studies and meta-analyses encompassing populations across numerous countries and patient populations consistently demonstrate that frail individuals experience higher rates of all-cause mortality, cardiovascular mortality, rehospitalization, and functional decline, even after adjusting for traditional cardiovascular risk factors [13,15,26]. This independence underscores frailty’s prognostic importance, as it is not simply present as a correlation to comorbidity but as a central determinant of outcome trajectories.

The prognostic weight of frailty becomes even more significant when considered in the context of cardiometabolic diseases (CMDs), such as ischemic heart diseases, diabetes, and stroke. There is mounting evidence that frailty and CMDs interact in an additive and, at times, synergistic fashion to accelerate adverse outcomes. For instance, patients with both frailty and cardiometabolic multimorbidity have notably higher risks of major adverse cardiac events and mortality when compared to those with either condition alone [21,22,28]. A prospective UK Biobank analysis revealed that frailty not only amplified the risks associated with CMDs but also altered their trajectory, accelerating progression to multimorbidity and raising long-term mortality risk [28]. Mendelian randomization studies further support a bidirectional relationship, where genetic predisposition to cardiometabolic disease can increase frailty risk and vice versa, although it should be noted that the aforementioned studies examined predominantly European populations [25]. These findings highlight the clinical reality that frailty magnifies the burden of existing cardiovascular pathology, creating a synergistic rather than merely additive effect on patient outcomes.

When examining prognostic accuracy, the choice of frailty assessment tool also plays an important role. The two dominant conceptual models described earlier (Fried’s frailty phenotype and the frailty index) have been validated across diverse populations [11,14]. The frailty phenotype, operationalized through observable criteria such as weakness, slowness, and low activity, provides a straightforward clinical tool and has demonstrated strong predictive validity for mortality and adverse cardiovascular outcomes [10,11,13,15]. In contrast, the frailty index, which calculates a ratio of accumulated health deficits ranging from comorbidities to functional impairments, offers a more granular and continuous measure of vulnerability [8]. Comparative studies suggest that while both instruments predict mortality, the phenotype often yields stronger effect sizes in relation to short-term outcomes, whereas the frailty index may better capture the spectrum of risk in prefrail and mildly frail individuals [21,28].

Taken together, frailty offers clinicians a key perspective through which to refine prognosis in cardiovascular care. By functioning as an independent predictor, interacting synergistically with CMDs, and varying in predictive strength depending on the assessment tool employed, frailty underscores the need for its integration into both clinical research and practice. Future models of risk prediction that fail to account for frailty are likely to underestimate true patient vulnerability, leaving clinicians ill-prepared to anticipate complications and appropriately tailor care.

## 6. Clinical Implications

The recognition of frailty as a key determinant of cardiovascular outcomes has important clinical implications, particularly as the population ages and the prevalence of multimorbidity increases. Integrating frailty assessment into cardiovascular practice provides clinicians with a more holistic understanding of patient vulnerability than traditional risk factors alone and has been shown by preliminary data to be cost-effective to implement into clinical practice [51]. Routine frailty assessment has the potential to refine risk prediction models, ensuring that therapeutic decisions reflect not only disease burden but also biological reserve and resilience [9]. Major barriers to widespread implementation of screening protocols include a shortage of medical personnel adequately trained in frailty assessment and management, as well as the time-consuming nature of the leading frailty assessment paradigms [52]. Single-metric screening tools such as grip strength, gait speed, or a 5 m walk test may be more practical to implement as widespread screening modalities to identify at-risk populations [6,10,42]. Although the International Conference of Frailty and Sarcopenia Research (ICFSR) has recommended that all adults aged 65 or older should be offered frailty screening through the use of rapid frailty instruments, uptake into mainstream societal guidelines remains sparse [53]. The American Academy of Family Physicians recommends frailty screening only in men aged 60 or older, women, current/former smokers, and patients with certain socioeconomic or metabolic risk factors, with just a “C” level evidence rating [54]. With increasing recognition of the bidirectional relationship between frailty and CVD, it is imperative that explicit recommendations around frailty screening and management be adopted by major preventive guidelines in future iterations.

Frailty assessment also plays a central role in decision-making for intensive therapies and cardiovascular procedures. Traditional approaches to procedural risk often rely on chronological age or comorbidity counts, which can overlook the heterogeneity in physiologic reserve among older adults. Patients of the same age may have vastly different tolerance for stressors such as surgery, percutaneous interventions, or chemotherapy, depending on their frailty status [8,13,15]. As previously discussed, studies have consistently demonstrated that frail individuals undergoing PCI or cardiac surgery experience significantly higher rates of perioperative complications, prolonged hospitalizations, and mortality compared to non-frail patients [43,44,46,48]. Recognizing frailty therefore has implications not only for procedural candidacy but also for discussions surrounding prognosis and goals of care [36]. In many cases, identifying frailty should prompt shared decision-making with patients and families, ensuring that treatment plans align with values and preferences, and that expectations for recovery are realistic. Moreover, frailty assessment can help determine when conservative management or palliative approaches may be more appropriate than aggressive interventions [6].

Beyond procedural decision-making, frailty influences the tailoring of both pharmacologic and lifestyle interventions. Polypharmacy is common in older adults with cardiovascular disease, yet frail individuals may be more susceptible to adverse drug reactions, drug–drug interactions, and functional decline as a result of medication burden [8]. Clinicians should therefore consider deprescribing where appropriate, prioritizing therapies that improve both survival and quality of life. Conversely, lifestyle interventions, including structured exercise, nutritional support, and weight management, are particularly important because frailty is increasingly recognized as a modifiable state. Evidence suggests that improvements in physical activity and dietary quality can not only slow the progression of frailty but also reduce the risk of cardiovascular disease over time [3,27]. For example, incorporating resistance training and mobility exercises into cardiac rehabilitation programs can restore strength and functional capacity, thereby enhancing resilience against future cardiovascular stressors [36]. Nutritional strategies, including adequate protein intake and anti-inflammatory dietary patterns, may further mitigate frailty progression while supporting cardiometabolic health [3,31]. Importantly, these interventions are most effective when individualized, reflecting the diverse physiologic and psychosocial needs of frail patients.

Taken together, the integration of frailty into cardiovascular practice shifts the paradigm from disease-centered to patient-centered care. Frailty assessment enhances prognostic accuracy, informs the appropriateness of invasive therapies, and promotes more personalized pharmacologic and lifestyle strategies. By acknowledging frailty as a modifiable and clinically actionable factor, cardiology can move toward more nuanced and compassionate care models that prioritize not only survival but also functional independence and quality of life. Future guidelines and care pathways should embed frailty assessment as a routine component of cardiovascular evaluation to ensure that patients receive treatment strategies aligned with both their medical risks and personal goals. Ultimately, this clinical recognition sets the stage for targeted interventions, ranging from exercise and nutrition programs to pharmacologic optimization, that aim to reverse or attenuate frailty and thereby improve cardiovascular outcomes.

## 7. Interventions Targeting Frailty and Cardiovascular Risk

Frailty is increasingly recognized as a modifiable condition, and many of the mechanisms that drive frailty—sarcopenia, inflammation, endothelial dysfunction, and impaired metabolic regulation—overlap with pathways central to CVD [16,29,31,32,33]. This shared biology means that interventions which strengthen physiologic reserve can meaningfully improve both frailty status and cardiovascular outcomes. Rather than being viewed as an irreversible manifestation of aging, frailty is better understood as a dynamic state responsive to targeted therapeutic strategies (Table 2) [8,9].

Lifestyle interventions form the foundation of frailty management. Exercise—particularly resistance and multicomponent training—has the strongest evidence base, improving muscle mass, mobility, and functional capacity, even among older adults with established frailty [10,11,12,36]. These gains translate into better cardiovascular health by enhancing endothelial function, reducing blood pressure, and improving lipid and glucose regulation [36]. The type of exercise conditioning matters, with outcomes differing between frail and non-frail adults: a recent meta-analysis encompassing 38 individual studies found multicomponent exercise to improve both physical function and mood, while strength training improved activities of daily living (ADL) performance in frail adults [55]. Similar beneficial effects were not seen with alternate exercise modalities such as aerobic or balance exercises, Tai Chi, and dancing [55]. Nutritional support further reinforces these benefits; protein-rich, anti-inflammatory dietary patterns counteract catabolic loss, promote muscle synthesis, and reduce cardiometabolic stress [3,31]. Weight management plays a complementary role, especially in sarcopenic obesity, where excess adiposity accelerates inflammation and disability. Sleep quality is an often overlooked determinant and also influences frailty trajectories through effects on metabolic regulation and inflammatory signaling [9]. Together, these lifestyle components provide a synergistic foundation for slowing or reversing frailty progression.

Optimizing cardiovascular risk factors is equally important. Frail individuals are more susceptible to hemodynamic instability, metabolic fluctuations, and adverse drug effects, making precise control of blood pressure, lipids, and glycemia essential [6,8]. Improvements in cardiometabolic parameters are associated with lower frailty incidence and slower progression toward multimorbidity [21,22,23,25,28]. Conversely, poor control of diabetes, metabolic syndrome, and obesity substantially increases frailty risk [23,25,26]. Although direct evidence for the efficacy of specific pharmacologic agents in reversing frailty is lacking, available indirect evidence suggests that the benefit of newer therapies such as SGLT2 inhibitors and GLP-1 receptor agonists in older adults with diabetes is proportional to the severity of frailty, likely due to direct correlations between frailty severity and metabolic dysfunction which renders frail adults at higher baseline risk of CVD and thus most likely to benefit from CVD risk factor optimization [56].

Because frailty involves multisystem impairment, multidisciplinary care models provide the most comprehensive and effective framework. Cardiac rehabilitation programs inherently combine supervised exercise, education, and risk factor optimization, offering improvements in both physical function and frailty status [36]. Collaboration between geriatrics and cardiology facilitates nuanced management of polypharmacy, cognition, functional limitations, and social support—domains central to frailty but often overlooked in cardiology practice [6]. Such integrated approaches ensure individualized, goal-concordant care plans that address both CVD and the broader determinants of physiological reserve.

A growing body of evidence confirms that frailty is modifiable. Longitudinal studies show that individuals frequently transition between frailty states, and targeted interventions can reverse or attenuate frailty progression [11,12,27]. Enhancing physical activity, nutritional status, and cardiometabolic health confers protective effects that persist across decades [32]. As mechanistic insights continue to evolve, intervention strategies are likely to become increasingly precise and impactful.

## 8. Conclusions

Frailty represents a powerful, multidimensional construct that captures the physiologic vulnerability underlying adverse cardiovascular outcomes. Across epidemiologic, mechanistic, and clinical domains, frailty consistently amplifies the burden of CVD by reducing resilience, accelerating functional decline, and heightening the risks associated with both medical and procedural therapies. Incorporating frailty assessment into cardiovascular care offers a more complete understanding of patient risk, enabling more precise prognostication and more patient-centered clinical decision-making. The growing body of evidence demonstrating that frailty is modifiable highlights the importance of early identification and targeted intervention, reinforcing frailty as a central consideration rather than a peripheral descriptor in cardiovascular medicine.

Looking ahead, advancing the field will require stronger evidence derived from longitudinal studies that capture frailty trajectories, deeper exploration of the biological mechanisms that link frailty with cardiovascular pathology, and more inclusive research that reflects sex- and race-specific differences in vulnerability. Equally important are clinical trials that directly evaluate frailty-targeted interventions in cardiovascular populations and the development of rapid, pragmatic screening tools that can be seamlessly integrated into busy clinical environments. Together, these efforts will help move the field toward more proactive, personalized, and equitable care, ultimately improving both cardiovascular outcomes and the overall well-being of older adults living with or at risk for frailty.

## Figures and Tables

**Table 1:** Comparison of the Two Conceptual Frameworks of Frailty.

Origin/Key Reference	Fried et al., 2001 [11] (Cardiovascular Health Study)	Rockwood & Mitnitski, 2001 [14] (Canadian Study of Health and Aging)
Conceptual Basis	Frailty as a biologic syndrome of decreased physiologic reserve and resistance to stressors	Frailty as an accumulation of health deficits across multiple systems
Measurement Approach	Uses a predefined set of physical indicators	Quantifies frailty by counting deficits across a broad range of variables
Common Criteria/Components	Unintentional weight loss, exhaustion, weakness, slow walking speed, low physical activity	Includes comorbidities, symptoms, signs, disabilities, and lab abnormalities (often 30+ variables)
Assessment Tool	Fried Frailty Phenotype (FFP)	Frailty Index (FI)
Scoring System	Categorizes individuals as robust, prefrail, or frail based on number of criteria present (0, 1–2, >3)	Provides a continuous score (ratio of deficits present + total deficits assessed)
Focus	Physical and performance-based domains	Global, multidimensional health deficits
Strengths	Simple, standardized, and predictive of adverse outcomes; clinically intuitive	Captures gradations of vulnerability and better discriminates between pre-frail and mildly frail states; highly predictive of mortality and institutionalization
Limitations	Narrow focus on physical function; may overlook cognitive and social dimensions; may underestimate frailty on the lower end of the frailty spectrum	Data-intensive; may be less practical for bedside use
Clinical Use	Often applied in geriatric or cardiovascular populations to stratify risk before interventions.	Used in epidemiologic studies and electronic health record-based frailty measurement

**Table 2:** Evidence-Based Recommendations for Frailty Interventions [53].

Recommendation	Quality of Evidence
Address polypharmacy, sarcopenia, reversible causes of weight loss, and causes of chronic fatigue	Very Low
Individuals with severe frailty should be referred to a geriatrician	No Data
Offer a multi-component physical activity program with a progressive, resistance-training component	Moderate
Protein/caloric supplementation should be paired with a physical activity program, especially for persons with frailty and concomitant weight loss or undernutrition	Low
Current pharmacologic therapies are not recommended for routine management of frailty	Low

Adapted from Dent E et al., 2019 [53].

## Data Availability

No new data were created or analyzed in this study. Data sharing is not applicable to this article.

## Abbreviations

ADL	Activities of Daily Living
CABG	Coronary Artery Bypass Graft
CKM	Cardiovascular–Kidney–Metabolic
CMD	Cardiometabolic Disease
CMM	Cardiometabolic Multimorbidity
CVD	Cardiovascular Disease
ICFSR	International Conference of Frailty and Sarcopenia Research
MACE	Major Adverse Cardiovascular Events
NSTEMI	Non-ST Elevation Myocardial Infarction
PCI	Percutaneous Coronary Intervention

## References

[1] Xi J.Y., Lin X., Hao Y.T. (2022). Measurement and projection of the burden of disease attributable to population aging in 188 countries, 1990–2050: A population-based study. J. Glob. Health.

[2] Global Burden of Metabolic Risk Factors for Chronic Diseases Collaboration (2014). Cardiovascular disease, chronic kidney disease, and diabetes mortality burden of cardiometabolic risk factors from 1980 to 2010: A comparative risk assessment. Lancet Diabetes Endocrinol..

[3] Soto M.E., Pérez-Torres I., Rubio-Ruiz M.E., Cano-Martínez A., Manzano-Pech L., Guarner-Lans V. (2023). Frailty and the Interactions between Skeletal Muscle, Bone, and Adipose Tissue-Impact on Cardiovascular Disease and Possible Therapeutic Measures. Int. J. Mol. Sci..

[4] Sato R., Vatic M., Peixoto da Fonseca G.W., Anker S.D., von Haehling S. (2024). Biological basis and treatment of frailty and sarcopenia. Cardiovasc. Res..

[5] Collard R.M., Boter H., Schoevers R.A., Oude Voshaar R.C. (2012). Prevalence of frailty in community-dwelling older persons: A systematic review. J. Am. Geriatr. Soc..

[6] Hoogendijk E.O., Afilalo J., Ensrud K.E., Kowal P., Onder G., Fried L.P. (2019). Frailty: Implications for clinical practice and public health. Lancet.

[7] Yang Y., Chen L., Filippidis F.T. (2025). Accelerometer-measured physical activity, frailty, and all-cause mortality and life expectancy among middle-aged and older adults: A UK Biobank longitudinal study. BMC Med..

[8] Kim D.H., Rockwood K. (2024). Frailty in Older Adults. N. Engl. J. Med..

[9] Howlett S.E., Rutenberg A.D., Rockwood K. (2021). The degree of frailty as a translational measure of health in aging. Nat. Aging.

[10] Fried L.P., Cohen A.A., Xue Q.L., Walston J., Bandeen-Roche K., Varadhan R. (2021). The physical frailty syndrome as a transition from homeostatic symphony to cacophony. Nat. Aging.

[11] Fried L.P., Tangen C.M., Walston J., Newman A.B., Hirsch C., Gottdiener J., Seeman T., Tracy R., Kop W.J., Burke G. (2001). Frailty in older adults: Evidence for a phenotype. J. Gerontol. A Biol. Sci. Med. Sci..

[12] Stenholm S., Ferrucci L., Vahtera J., Hoogendijk E.O., Huisman M., Pentti J., Lindbohm J.V., Bandinelli S., Guralnik J.M., Kivimäki M. (2019). Natural Course of Frailty Components in People Who Develop Frailty Syndrome: Evidence from Two Cohort Studies. J. Gerontol. A Biol. Sci. Med. Sci..

[13] Xue Q.L., Bandeen-Roche K., Tian J., Kasper J.D., Fried L.P. (2021). Progression of Physical Frailty and the Risk of All-Cause Mortality: Is There a Point of No Return?. J. Am. Geriatr. Soc..

[14] Mitnitski A.B., Mogilner A.J., Rockwood K. (2001). Accumulation of deficits as a proxy measure of aging. ScientificWorldJournal.

[15] Damluji A.A., Chung S.E., Xue Q.L., Hasan R.K., Moscucci M., Forman D.E., Bandeen-Roche K., Batchelor W., Walston J.D., Resar J.R. (2021). Frailty and cardiovascular outcomes in the National Health and Aging Trends Study. Eur. Heart J..

[16] Veronese N., Custodero C., Cella A., Demurtas J., Zora S., Maggi S., Barbagallo M., Sabbà C., Ferrucci L., Pilotto A. (2021). Prevalence of multidimensional frailty and pre-frailty in older people in different settings: A systematic review and meta-analysis. Ageing Res. Rev..

[17] Blodgett J., Theou O., Kirkland S., Andreou P., Rockwood K. (2015). Frailty in NHANES: Comparing the Frailty Index and Phenotype. Arch. Gerontol. Geriatr..

[18] Davidson S.L., Lee J., Emmence L., Bickerstadd E., Rayers G., Davidson E., Richardson J., Anderson H., Walker R., Dotchin C. (2025). Systematic Review and Meta-Analysis of the Prevalence of Frailty and Pre-Frailty Amongst Older Hospital Inpatients in Low- and Middle-Income Countries. Age Ageing.

[19] O’Caoimh R., Sezgin D., O’Donovan M.R., Molloy D.W., Clegg A., Rockwood K., Liew A. (2021). Prevalence of frailty in 62 countries across the world: A systematic review and meta-analysis of population-level studies. Age Ageing.

[20] Gordon E.H., Peel N.M., Samanta M., Theou O., Howlett S.E., Hubbard R.E. (2017). Sex differences in frailty: A systematic review and meta-analysis. Exp. Gerontol..

[21] Gao K., Li B.L., Yang L., Zhou D., Ding K.X., Yan J., Gao Y.J., Huang X.R., Zheng X.P. (2021). Cardiometabolic diseases, frailty, and healthcare utilization and expenditure in community-dwelling Chinese older adults. Sci. Rep..

[22] Zhou K., Wang A., Yi K. (2025). Cardiometabolic multimorbidity and frailty in middle-aged and older adults: A cross-nationally harmonized study. Front. Public Health.

[23] McCarthy K., Laird E., O’Halloran A.M., Fallon P., Ortuño R.R., Kenny R.A. (2023). Association between metabolic syndrome and risk of both prevalent and incident frailty in older adults: Findings from The Irish Longitudinal Study on Ageing (TILDA). Exp. Gerontol..

[24] Huang Q., Luo X., Yang T., Qing X., Zhang X., Xiu M., Liu Z., Li H., Liu S., Xiao W. (2025). Longitudinal changes in frailty, insulin resistance and incident cardiovascular-kidney- metabolic syndrome progression in middle-aged and older adults: Evidence from CHARLS. Diabetol. Metab. Syndr..

[25] Zhu J., Zhou D., Wang J., Yang Y., Chen D., He F., Li Y. (2022). Frailty and cardiometabolic diseases: A bidirectional Mendelian randomisation study. Age Ageing.

[26] Liu X., Tou N.X., Gao Q., Gwee X., Wee S.L., Ng T.P. (2022). Frailty and risk of cardiovascular disease and mortality. PLoS ONE.

[27] He D., Wang Z., Li J., Yu K., He Y., He X., Liu Y., Li Y., Fu R., Zhou D. (2024). Changes in frailty and incident cardiovascular disease in three prospective cohorts. Eur. Heart J..

[28] Ma T., He L., Luo Y., Fu D., Huang J., Zhang G., Cheng X., Bai Y. (2023). Frailty, an Independent Risk Factor in Progression Trajectory of Cardiometabolic Multimorbidity: A Prospective Study of UK Biobank. J. Gerontol. A Biol. Sci. Med. Sci..

[29] Stewart R. (2019). Cardiovasc. Dis. Frailty: What Are Mech. Links?. Clin. Chem..

[30] Veronese N. (2020). Frailty as Cardiovascular Risk Factor (and Vice Versa). Adv. Exp. Med. Biol..

[31] Soysal P., Arik F., Smith L., Jackson S.E., Isik A.T. (2020). Inflammation, Frailty and Cardiovascular Disease. Adv. Exp. Med. Biol..

[32] Barbalho S.M., Tofano R.J., Chagas E.F.B., Detregiachi C.R.P., de Alvares Goulart R., Flato U.A.P. (2021). Benchside to the bedside of frailty and cardiovascular aging: Main shared cellular and molecular mechanisms. Exp. Gerontol..

[33] Amarasekera A.T., Chang D., Schwarz P., Tan T.C. (2021). Does vascular endothelial dysfunction play a role in physical frailty and sarcopenia? A systematic review. Age Ageing.

[34] Piechocki M., Przewlocki T., Pieniazek P., Trystula M., Podolec J., Kablak-Ziembicka A. (2024). A Non-Coronary, Peripheral Arterial Atherosclerotic Disease (Carotid, Renal, Lower Limb) in Elderly Patients—A Review PART II—Pharmacological Approach for Management of Elderly Patients with Peripheral Atherosclerotic Lesions outside Coronary Territory. J. Clin. Med..

[35] Radu A.F., Radu A., Bungau G.S., Tit D.M., Vesa C.M., Jurca T., Uivarosan D., Gitea D., Brata R., Bustea C. (2026). Polypharmacy and Drug–Drug Interaction Architecture in Hospitalized Cardiovascular Patients: Insights from Real-World Analysis. Biomedicines.

[36] Forman D.E., Arena R., Boxer R., Dolansky M.A., Eng J.J., Fleg J.L., Haykowsky M., Jahangir A., Kaminsky L.A., Kitzman D.W. (2017). Prioritizing Functional Capacity as a Principal End Point for Therapies Oriented to Older Adults with Cardiovascular Disease: A Scientific Statement for Healthcare Professionals From the American Heart Association. Circulation.

[37] Gordon E.H., Hubbard R.E. (2019). Do sex differences in chronic disease underpin the sex-frailty paradox?. Mech. Ageing Dev..

[38] Park C., Ko F.C. (2021). The Science of Frailty: Sex Differences. Clin. Geriatr. Med..

[39] He Y.Y., Chang J., Wang X.J. (2022). Frailty as a predictor of all-cause mortality in elderly patients undergoing percutaneous coronary intervention: A systematic review and meta-analysis. Arch. Gerontol. Geriatr..

[40] Shrauner W., Lord E.M., Nguyen X.T., Song R.J., Galloway A., Gagnon D.R., Driver J.A., Gaziano J.M., Wilson P.W.F., Djousse L. (2022). Frailty and cardiovascular mortality in more than 3 million US Veterans. Eur. Heart J..

[41] Zhu C., Ma T., He L., Cheng X., Bai Y. (2025). Joint Associations of Frailty and Cardiometabolic Diseases with Risk of All-Cause and Cardiac Mortality. JACC Asia.

[42] Tang K.S., Fan W. (2025). Beyond the Sum of Their Parts: Frailty and Cardiometabolic Disease in Predicting Mortality. JACC Asia.

[43] Wong C.W.Y., Yu D.S.F., Li P.W.C., Chan B.S. (2023). The prognostic impacts of frailty on clinical and patient-reported outcomes in patients undergoing coronary artery or valvular surgeries/procedures: A systematic review and meta-analysis. Ageing Res. Rev..

[44] Wang P., Zhang S., Zhang K., Tian J. (2021). Frailty Predicts Poor Prognosis of Patients After Percutaneous Coronary Intervention: A Meta-Analysis of Cohort Studies. Front. Med..

[45] Ekerstad N., Swahn E., Janzon M., Alfredsson J., Löfmark R., Lindenberger M., Carlsson P. (2011). Frailty is independently associated with short-term outcomes for elderly patients with non-ST-segment elevation myocardial infarction. Circulation.

[46] Kumar S., Kearney K.E., Chung C.J., Elison D., Steinberg Z.L., Lombardi W.L., McCabe J.M., Azzalini L. (2025). Frailty and Cardiovascular Outcomes in Patients Undergoing Percutaneous Coronary Intervention. Catheter. Cardiovasc. Interv..

[47] Tse G., Gong M., Nunez J., Sanchis J., Li G., Ali-Hasan-Al-Saegh S., Wong W.T., Wong S.H., Wu W.K.K., Bazoukis G. (2017). Frailty and Mortality Outcomes After Percutaneous Coronary Intervention: A Systematic Review and Meta-Analysis. J. Am. Med. Dir. Assoc..

[48] Sepehri A., Beggs T., Hassan A., Rigatto C., Shaw-Daigle C., Tangri N., Arora R.C. (2014). The impact of frailty on outcomes after cardiac surgery: A systematic review. J. Thorac. Cardiovasc. Surg..

[49] Kwok C.S., Lundberg G., Al-Faleh H., Sirker A., Van Spall H.G.C., Michos E.D., Rashid M., Mohamed M., Bagur R., Mamas M.A. (2019). Relation of Frailty to Outcomes in Patients with Acute Coronary Syndromes. Am. J. Cardiol..

[50] Nguyen D.D., Arnold S.V. (2023). Impact of frailty on disease-specific health status in cardiovascular disease. Heart.

[51] Li Z., Habbous S., Thain J., Hall D.E., Nagpal D., Bagur R., Kiaii B., John-Baptiste A. (2022). Cost-Effectiveness Analysis of Frailty Assessment in Older Patients Undergoing Coronary Artery Bypass Grafting (CABG) Surgery. Can. J. Cardiol..

[52] Damjanac T., Lynch D.H., Spangler H., Shah R.R., Mournighan K., Gao M., Zeng D., Batsis J.A. (2025). Measuring Frailty in Clinical Practice: Overcoming Challenges with Implementation. J. Am. Geriatr. Soc..

[53] Dent E., Morley J.E., Cruz-Jentoft A.J., Woodhouse L., Rodríguez-Mañas L., Fried L.P., Woo J., Aprahamian I., Sanford A., Lundy J. (2019). Physical Frailty: ICFSR International Clinical Practice Guidelines for Identification and Management. J. Nutr. Health Aging.

[54] Allison R., Assadzandi S., Adelman M. (2021). Frailty: Evaluation and Management. Am. Fam. Physician.

[55] Alowaydhah S., Weerasekara I., Walmsley S., Marquez J. (2024). Physical Exercise for Healthy Older Adults and Those with Frailty: What Exercise is Best and is There a Difference? A Systematic Review and Meta-Analysis. Curr. Gerontol. Geriatr. Res..

[56] Sinclair A.J., Abdelhafiz A.H. (2025). The Use of SGLT-2 Inhibitors and GLP-1RA in Frail Older People with Diabetes: A Personalised Approach Is Required. Metabolites.

